# NAD+ metabolism at the host–virus interface

**DOI:** 10.1099/jgv.0.002285

**Published:** 2026-06-22

**Authors:** Prince Jhandai, Kishore Vaddadi, Lin Liu

**Affiliations:** 1 Oklahoma Center for Respiratory and Infectious Diseases, Oklahoma State University, Stillwater, OK, USA; 2 The Lundberg-Kienlen Lung Diseases and Infection Laboratory, Department of Physiological Sciences, Oklahoma State University, Stillwater, OK, USA

**Keywords:** CD38, coronavirus, influenza A virus, nicotinamide adenine dinucleotide (NAD^+^) metabolism, poly (ADP-ribose) polymerases (PARPs), sirtuins

## Abstract

Nicotinamide adenine dinucleotide (NAD^+^) is one of the most important metabolic coenzymes that not only drives redox reactions and energy production but also acts as a critical substrate for several enzymes involved in immune signalling, DNA repair and epigenetic regulation. Viral infections are known as potent modulators of NAD^+^ metabolism, with pathogens such as SARS-CoV-2, influenza A virus, Zika virus, herpes simplex virus and human immunodeficiency virus altering NAD^+^ biosynthesis and consumption to benefit their persistence and replication. In this review, we summarize the current understanding of NAD^+^ metabolism and its regulatory enzymes: sirtuins, poly (ADP-ribose) polymerases and CD38/CD157. We then discuss the interplay between NAD^+^ homeostasis and virus infection. Understanding how diverse viruses manipulate NAD^+^ metabolism could lead to broad-spectrum antiviral strategies grounded in metabolic resilience.

## Introduction

Viral infections have posed significant global health threats and challenges, with seasonal outbreaks and major pandemics such as the 1918 influenza pandemic and the recent COVID-19 crisis causing widespread impact across species. These events highlight the urgent need to deepen our understanding of viral life cycles and host–virus interactions. Devoid of any locomotion and metabolic activities, many viruses rely on host metabolism, including pathways critical for energy production, immune signalling and biosynthesis to complete their life cycle [1,2].

Nicotinamide adenine dinucleotide (NAD^+^) is a cofactor or coenzyme and plays a crucial role in maintaining cellular metabolism and cell survival. NADH, the reduced form of NAD^+^, is produced by numerous enzymes during glycolysis and the Krebs cycle and is later oxidized back to NAD^+^ during oxidative phosphorylation, thereby enabling ATP generation [3]. NAD^+^ can also act as a substrate for a diverse group of enzymes, including poly (ADP-ribose) polymerases (PARPs), sirtuins and CD38. These enzymes play essential roles in several aspects of cellular homeostasis, and their NAD^+^-consuming activities have been implicated in shaping host–virus interactions. In many cases, they can also be co-opted by viruses to facilitate infection and replication [4].

Many recent studies have shown complex roles of these enzymes during viral infection ranging from directly binding to viral proteins to promoting IFN responses and modulating inflammatory damage. However, despite growing evidence, the broader picture of how NAD^+^ metabolism contributes to or restricts viral pathogenesis remains incomplete. In this review, we aim to summarize current knowledge on the role of NAD^+^ metabolism and its key regulators during viral infections, with a particular focus on viruses, including SARS-CoV-2, influenza A virus (IAV), Zika virus (ZIKV), herpes simplex virus (HSV) and human immunodeficiency virus (HIV). We discuss how viruses alter NAD^+^ homeostasis, the dual roles of NAD^+^-consuming enzymes in immunity and viral replication and the emerging therapeutic potential of NAD^+^-modulating compounds.

## NAD^+^ metabolism

NAD^+^ exists in two forms: NAD^+^ and its reduced form after accepting a hydride ion: NADH; both forms are central to the metabolism of all major biomolecules. NAD^+^ transfers energy between different metabolic pathways such as the Krebs cycle and fatty acid oxidation by accepting electrons, and NADH, on the other hand, is oxidized during glycolysis and oxidative phosphorylation for ATP production [5,6]. NAD^+^ can be converted into many key molecules that are important for cell signalling and energy transductions, and some of these molecules are nicotinamide adenine dinucleotide phosphate (NADP), cyclic ADP-ribose (cADPr) and nicotinic acid adenine dinucleotide phosphate [7].

**Fig. 1.**
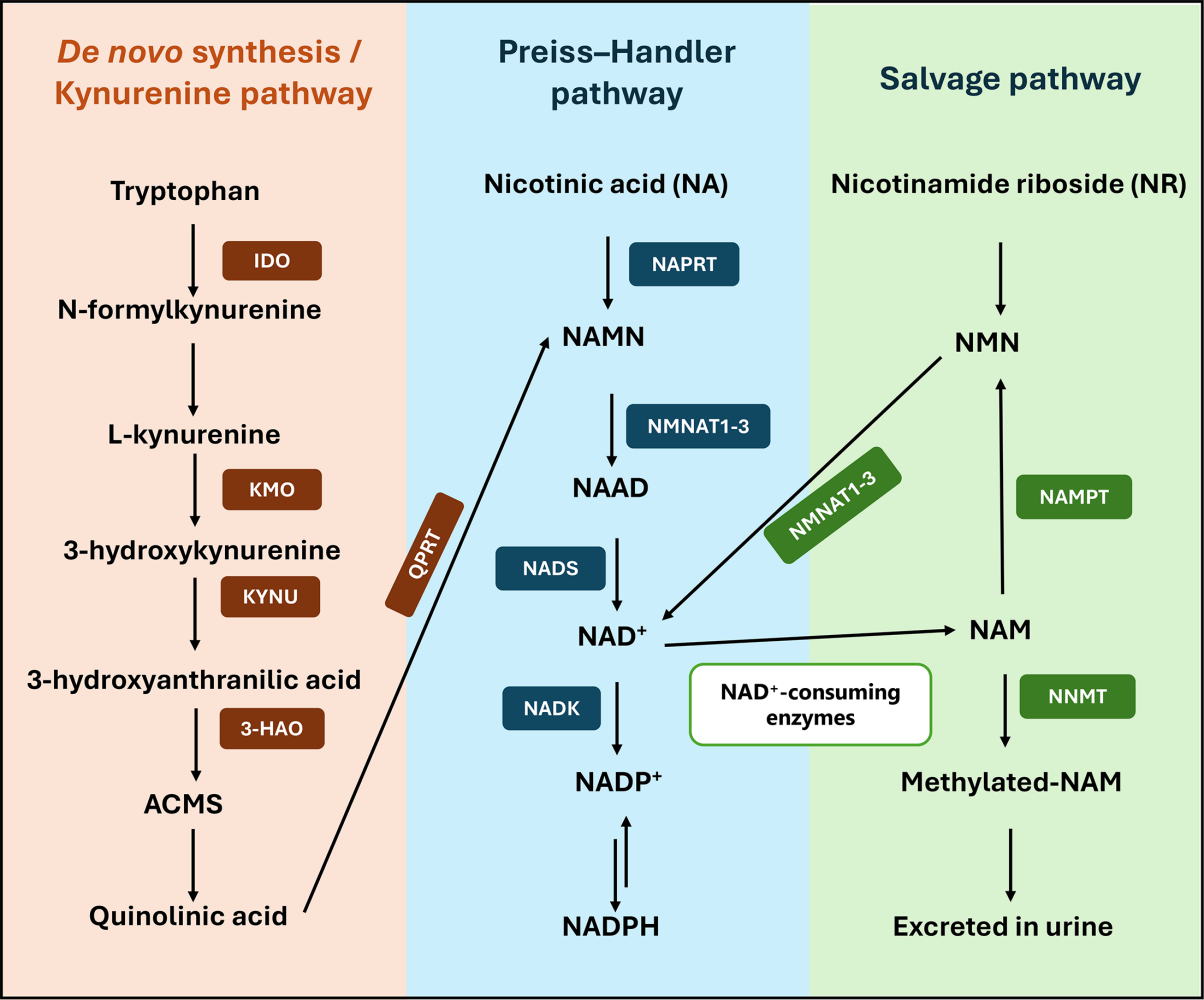
Overview of NAD^+^ metabolism. NAD^+^ is synthesized via three major pathways: the *de novo* (kynurenine) pathway, the Preiss–Handler pathway and the salvage pathway. In these pathways, intermediates including NAMN and NMN are generated from tryptophan, nicotinic acid and nicotinamide riboside by various enzymes and then are converted to NAD^+^ by NMNATs and/or NADS. NAD^+^ can be phosphorylated to NADP^+^ and reduced to NADPH or regenerated from NAM via NAMPT. Excess NAM may be methylated by NNMT for excretion. Abbreviations: ACMS, *α*-amino *β*-carboxymuconate *ε*-semialdehyde; NAMN, nicotinate mononucleotide; NAAD, nicotinate adenine dinucleotide; NAM, nicotinamide; NMN, nicotinamide mononucleotide; IDO, indoleamine 2,3-dioxygenase; KMO, kynurenine 3-monooxygenase; KYNU, kynureninase; 3-HAO, 3-hydroxyanthranilate 3,4-dioxygenase; QPRT, quinolinate phosphoribosyltransferase; NAPRT, nicotinic acid phosphoribosyltransferase; NMNAT1-3, nicotinamide mononucleotide adenylyltransferase 1–3; NADS, NAD^+^ synthetase; NADK, NAD kinase; NAMPT, nicotinamide phosphoribosyltransferase; NNMT, nicotinamide *N*-methyltransferase.

NAD^+^ levels in any eukaryotic cell are mainly dependent on NAD^+^ biosynthesis and NAD^+^ degradation. There are three major pathways that are responsible for maintaining NAD^+^ levels: *de novo* biosynthesis, Preiss–Handler pathway and salvage pathway [7,8] (Fig. 1).

The *de novo* synthesis or kynurenine (KYNU) pathway uses tryptophan, a dietary amino acid for the formation of NAD^+^. Tryptophan enters the cells via distinct types of transporters of solute carrier family such as SLC7A5, SLC36A4, SLC7A8, SLC16A10 and SLC6A19 and is converted into *N-*formylkynurenine by the rate-limiting enzyme indoleamine 2,3-dioxygenase [9]. *N-*Formylkynurenine is further transformed into l-KYNU, which converts into 3-hydroxykynurenine by KYNU 3-monooxygenase and then 3-hydroxyanthranilic acid (3-HAA) by KYNU. Next, 3-HAA is turned into *α*-amino-*β*-carboxymuconate *ε*-semialdehyde (ACMS) by 3-hydroxyanthranilate 3,4-dioxygenase. Finally, ACMS spontaneously cyclizes to form quinolinic acid, which acts as a precursor for the formation of nicotinic acid mononucleotide (NAMN) by quinolinate phosphoribosyl transferase. NAMN acts as a link between the *de novo* pathway with the Preiss–Handler pathway [7,10].

The Preiss–Handler pathway is a three-step process that begins with nicotinic acid (NA). NA enters the cell via transporters like SLC5A8, SLC22A7 and SLC22A13. Nicotinic acid phosphoribosyltransferase (NAPRT) converts NA into NAMN, which is then transformed into nicotinic acid adenine dinucleotide (NAAD) by nicotinamide mononucleotide adenylyl transferases 1–3 (NMNAT1-3). Finally, NAD^+^ synthetase completes the pathway by transforming NAAD into NAD^+^ for cellular use [7,10].

In the salvage pathway, cellular NAD^+^ levels are maintained through the degradation and consumption of NAD^+^ by NAD^+^-consuming enzymes like PARPs and sirtuins, which generate nicotinamide (NAM). NAM is then recycled back to NAD^+^: it is first transformed into nicotinamide mononucleotide (NMN) by nicotinamide phosphoribosyl transferase (NAMPT) and subsequently converted into NAD^+^ via the NMNAT1-3, the same enzymes for converting NAMN to NAAD. NAM can also be methylated by nicotinamide *N*-methyltransferase and excreted in the urine, thereby preventing NAD^+^ recycling [7,10].

In addition to NAM, NAD^+^ can also be synthesized from other precursors such as nicotinamide riboside (NR) via the salvage pathway, where nicotinamide riboside kinases (NRK1 and NRK2) phosphorylate NR to NMN. This pathway supports efficient reutilization of precursors, conserves cellular resources and ensures a continuous NAD^+^ supply, particularly under conditions such as metabolic stress or ageing, when NAD^+^ levels decline. NAD^+^ can also be reduced to NADH or phosphorylated by NAD kinase to form NADP^+^, which can then be reduced to NADPH [7,10].

## NAD^+^-consuming enzymes

**Fig. 2.**
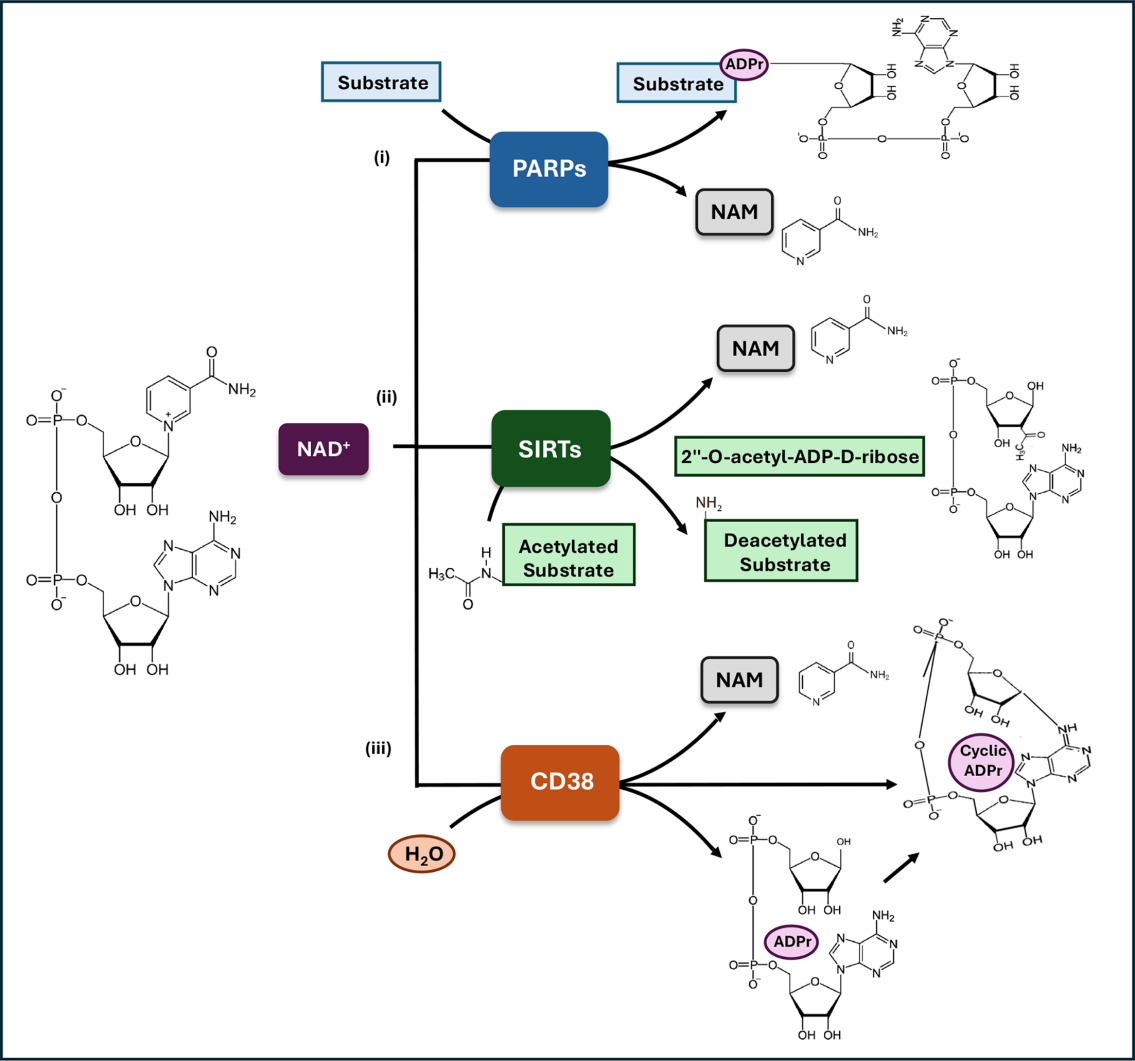
NAD-consuming enzymes. AD^+^ serves as a substrate for several enzyme families that regulate metabolism, signalling and stress responses. PARPs transfer ADP-ribose (ADPr) from NAD^+^ to proteins, producing mono- or poly-ADP-ribosylated substrates and NAM. Sirtuins deacetylate proteins in an NAD^+^-dependent manner, producing deacetylated proteins, NAM and 2′-O-acetyl-ADP-d-ribose. CD38/CD157 hydrolyse NAD^+^ to generate ADPr, cADPr and NAM, modulating calcium signalling and immune responses.

NAD^+^ consumption by different enzymes is one of the most important parts of the eukaryotic host system to maintain the dynamics of various proteins. NAD^+^ is utilized by three major categories of enzymes (Fig. 2): (i) PARPs, which are enzymes that catalyse the transfer of ADP-ribosyl group from NAD^+^ to target proteins, (ii) sirtuins, which are NAD^+^-dependent deacetylases and ADP-ribosyl transferases, and (iii) CD38 and CD157, which are ectoenzymes, membrane proteins with their catalytic sites located outside the cells, and hydrolyse NAD^+^ to ADP ribose and NAM [4].

### PARPs

PARPs are a family of enzymes primarily involved in DNA damage repair. They also play roles in other processes such as chromatin remodelling, transcriptional regulation and cell death pathways [11,13]. The human PARP family is composed of 17 proteins, classified based on poly or mono (ADP-ribosyl) polymerase activity. The primary function of some of PARPs, particularly PARP1 and PARP2, is to detect DNA damage, bind DNA strand breaks and use NAD^+^ as a substrate to synthesize poly and mono (ADP-ribose) chains. These chains are covalently attached to PARPs themselves and other target proteins such as histones and DNA repair factors, including XRCC1, serving as a post-translational modification that facilitates the recruitment of DNA repair complexes to sites of damage. The activity of PARPs requires a significant amount of NAD^+^, which links cellular NAD^+^ levels to the DNA repair process. PARP1 is the most studied member of the family, accounting for ~90% of total PARP activity in response to DNA damage.

### Sirtuins

Sirtuins have diverse cellular functions, including ageing, apoptosis, inflammation, transcription and stress resistance [14,15]. The sirtuin family is composed of seven proteins, each with distinct subcellular localizations: SIRT1 and SIRT6 are present in the nucleus; SIRT7 in the nucleolus; SIRT1, SIRT2 and SIRT5 in the cytoplasm; and SIRT3, SIRT4 and SIRT5 in the mitochondria. Their localization patterns emphasize how changes in intracellular NAD^+^ pools can selectively impact organelle-specific sirtuin functions. Sirtuins are constitutively active in cells, with SIRT1 and SIRT2 responsible for roughly one third of the total NAD^+^ consumption under normal physiological conditions. Sirtuins use NAD^+^ as a substrate to remove acetyl groups from lysine residues on target proteins, thereby altering their activity, localization and interactions. This deacetylation reaction consumes NAD^+^ and generates NAM and O-acetyl-ADP-ribose as by-products. Through these actions, sirtuins contribute to metabolic regulation, mitochondrial function and genomic stability.

### CD38 and CD157

CD38 and CD157 (BST-1) are membrane-associated ADP-ribosyl cyclases; CD38 is a type II transmembrane glycoprotein, while CD157 is mainly GPI-anchored on the cell surface. These enzymes convert NAD^+^ into the calcium-mobilizing messengers cADPR and ADPR, which promote Ca²^+^ release from the endoplasmic reticulum. This calcium signalling helps regulate essential physiological processes, including insulin secretion, smooth and skeletal muscle contraction and the activation of immune cells that rely on calcium-dependent pathways. CD38 and CD157 are also known as ADP-ribosyl cyclase 1 and ADP-ribosyl cyclase 2, respectively, reflecting their enzymatic activities [16,17].

CD38 additionally influences metabolic regulation, mitochondrial function and energy homeostasis through NAD^+^ consumption. In the immune system, CD38 functions as both an enzyme and a receptor, contributing to leucocyte activation, migration, adhesion and cytokine production [18,19]. Compared to CD38, the receptor functions of CD157 are less well characterized. However, several studies suggest that CD157 activation by specific monoclonal antibodies, such as RF3, Bec-7, SG2 and Mo5, promotes the trafficking of neutrophils and monocytes [20].

## NAD^+^ metabolism and virus infection

Viruses rely completely on the host cell machinery to complete their life cycle, functioning as obligatory intracellular parasites. To replicate, they hijack and reprogram various host processes to meet their own needs. In turn, the host mounts immune defences to combat viral invasion, turning infected cells into battlegrounds where viruses and the immune system engage in a dynamic struggle [1,2]. Since NAD^+^ metabolism is central to cellular energy production, redox balance and key signalling pathways, many viruses have evolved mechanisms to manipulate host NAD^+^ levels and metabolic pathways to support their survival and replication. This interplay highlights the complex relationship between viral infection and host metabolic regulation. In this section, we summarize several examples of viruses such as coronaviruses, influenza virus, HIV, ZIKV and HSV that employ distinct mechanisms to alter and respond to NAD^+^ metabolism.

### Coronaviruses

**Fig. 3.**
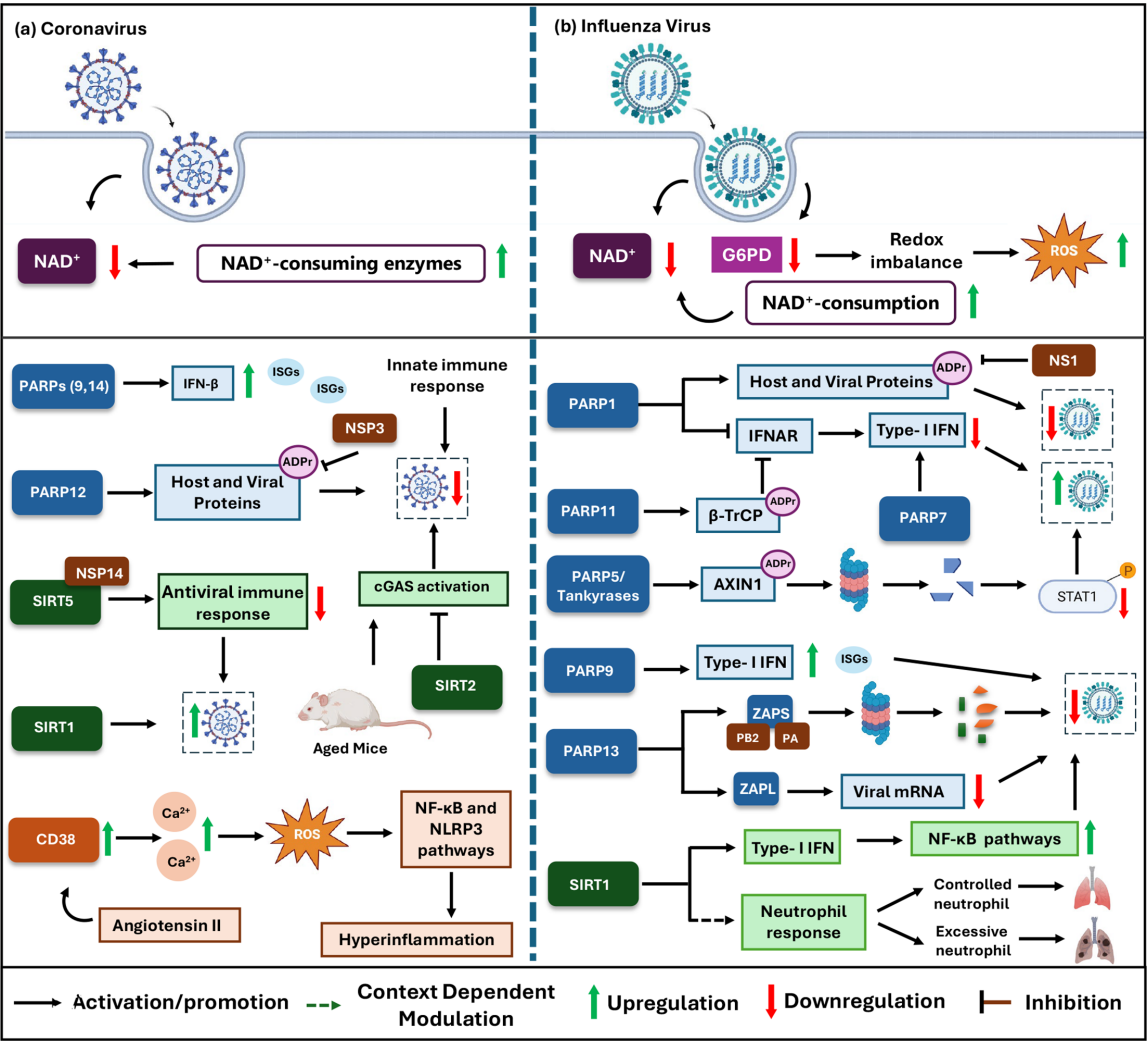
NAD^+^ metabolism and viral responses during coronavirus and influenza virus infection. (**a**) Coronavirus infection: viral entry reduces cellular NAD^+^ levels through an increase in NAD^+^-consuming enzymes, including PARP9, 12 and 14; SIRT1 and 5; and CD38. Viral NSP14 interacts with SIRT5, influencing host antiviral signalling. SIRT2 activity modulates the cGAS-STING pathway and contributes to antiviral defence. NAD^+^ depletion contributes to altered calcium signalling via CD38, leading to reactive oxygen species (ROS) production. ROS and NAD^+^ modulation affect NF-κB and NLRP3 inflammasome pathways, resulting in changes to IFN-β production. Overall, coronavirus infection leads to complex host NAD^+^ alterations, oxidative stress and immune signalling changes. (**b**) Influenza virus entry similarly reduces NAD^+^ levels through enhancing NAD^+^ consumption and impairs glucose-6-phosphate dehydrogenase (G6PD) activity, resulting in NADPH depletion and increased ROS accumulation. PARP1 has a context-dependent role. It inhibits IFNAR resulting in decreased IFN response, helping viruses, and it also mediates ADP-ribosylation of host and viral proteins and exerts antiviral effects. However, the viral NS1 protein antagonizes PARP1-mediated ADP-ribosylation. PARP11 negatively regulates type I IFN (IFN-I) signalling through ADP-ribosylation–dependent modulation of *β*-TrCP, leading to reduced IFNAR stability and dampened downstream signalling. PARP7 also functions as a suppressor of IFN-I production, limiting antiviral responses. Tankyrase-mediated ADP-ribosylation of AXIN1 promotes its proteasomal degradation, contributing to proviral signalling through suppression of IFN-1 responses and STAT1 activity. In contrast, PARP9 acts as a positive regulator of IFN signalling by enhancing IFN-stimulated gene (ISG) expression, thereby promoting antiviral defence. PARP13, ZAPS and ZAPL restrict influenza virus replication by targeting viral proteins (PB2 and PA) and viral mRNA for degradation. SIRT1 modulates IFN-I signalling and neutrophil responses, influencing NF-κB pathways and antiviral defence in the lung. Abbreviations: ROS, reactive oxygen species; NAD^+^, nicotinamide adenine dinucleotide; PARPs, poly (ADP-ribose) polymerases; SIRT, sirtuins; NF-κB, nuclear factor kappa-light-chain-enhancer of activated B cells; NLRP3, NOD-like receptor family pyrin domain containing 3; cGAS, cyclic GMP-AMP synthase.

Coronaviruses are enveloped, positive-sense single-stranded RNA viruses that infect humans and animals and cause systemic respiratory diseases [21,22]. Highly pathogenic coronaviruses, including SARS-CoV-2, SARS-CoV and MERS-CoV, are associated with severe lung pathology and high mortality. The coronavirus genome encodes both structural proteins and multiple non-structural proteins that assemble the replication–transcription complex and actively modulate host cellular and immune pathways to support viral replication and pathogenesis (Fig. 3a).

#### Infection-induced alterations in NAD^+^ metabolism

Clinical data demonstrate that hospitalized COVID-19 patients exhibit an altered NAD^+^ metabolome. Despite only modest reductions in absolute NAD^+^ levels, increased NAD^+^ turnover and accumulation of NAD^+^ degradation products (e.g. 1-methylnicotinamide) are observed, alongside transcriptional changes in NAD^+^-dependent pathways such as DNA repair, redox homeostasis and mitochondrial function [23]. SARS-CoV-2 infection alters host NAD^+^ metabolism by upregulating genes involved in the salvage pathway, including NAMPT and NRK1/2, while downregulating *de novo* NAD^+^ biosynthetic pathways [24]. Data mining of publicly available transcriptomic datasets further showed that NAD^+^ synthesis genes, including *NAPRT1*, *NMNAT1*, *NRK* and indoleamine 2,3-dioxygenase 1 (*IDO1*), were upregulated in the lungs of patients who died from COVID-19 infection [25]. These changes were also observed in the lungs of SARS-CoV-2-infected mice [26].

Coronavirus infection also alters host NAD^+^ metabolism by increasing expression of NAD^+^-consuming enzymes, including multiple PARPs, such as PARP9, PARP10 and PARP14, which consume NAD^+^ to carry out ADP-ribosylation reactions [24]. Overactivation of PARPs during infection can reduce intracellular NAD^+^ levels, potentially impacting cellular metabolism and immune functions. Supplementation with NAD^+^ precursors restores NAD^+^ pools [24,27, 28].

A recent study showed that the SARS-CoV-2 Spike protein can directly inhibit lactate dehydrogenase B (LDHB), an enzyme that performs the NAD^+^-dependent conversion of lactate to pyruvate [29]. Suppression of LDHB leads to lactate accumulation and drives a shift towards anaerobic glycolysis, revealing an additional way in which SARS-CoV-2 disrupts cellular metabolic homeostasis and NAD^+^-linked reactions.

Dysregulation of NAD^+^ metabolism impairs immune functions. In patients with COVID-19, plasma levels of several NAD^+^-related metabolites such as NAD^+^ precursor NMN and NAM are significantly lower than in healthy controls, coinciding with elevated inflammatory cytokines, including IL-6, IL-8, IL-10, IL-1α and macrophage colony-stimulating factor [30]. These observations suggest that NAD^+^ depletion is linked to the hyperinflammatory response observed in severe COVID-19. SARS-CoV-2 can also deplete mitochondrial NAD^+^, impair IFN responses and thereby compromise innate immunity [31]. Disrupted NAD^+^ pathways are also implicated in post-acute COVID-19 syndrome, where persistent NAD^+^ depletion may underlie chronic fatigue and immune dysregulation [32].

Most research on NAD^+^ metabolism has focused on SARS-CoV-2, but one study showed that the MERS-CoV macrodomain can bind not only ADPr but also NAD^+^ and other related metabolites, with binding affinity increasing at physiological temperature, suggesting adaptation to fluctuations in host NAD^+^ levels [33].

#### Impact of NAD^+^-consuming enzymes on infection

NAD^+^-consuming enzymes, including the PARP family, sirtuins and CD38, play critical roles in coronavirus infection and host responses. PARPs mediate ADP-ribosylation of viral and host proteins, a post-translational modification that can restrict or promote viral replication. Coronaviruses encode a conserved macrodomain within nonstructural protein 3 (Nsp3), referred to as the coronavirus ADP-ribosylhydrolase, which plays a critical role in counteracting host antiviral ADP-ribosylation. Studies in mouse models demonstrated that macrodomain catalytic activity is essential for virulence. Viruses carrying mutations in this domain show attenuated replication, reduced inflammation and enhanced IFN responses [34,35]. Biochemical analyses further revealed that coronavirus macrodomains from SARS-CoV, MERS-CoV and other positive-sense RNA viruses efficiently remove mono-ADPr, directly reversing PARP-mediated antiviral modifications [36]. SARS-CoV-2 Mac1 (the Nsp3 macrodomain) is essential for viral replication and immune evasion, as loss of Mac1 activity leads to impaired virus replication and enhanced IFN-1 responses in both cell culture systems and mouse models, demonstrating its critical role as an IFN antagonist [37]. The SARS-CoV-2 Nsp3 macrodomain also fights host antiviral defences by hydrolysing ADP-ribosylation catalysed by the IFN-inducible PARP9/deltex E3 ubiquitin ligase 3-like protein (DTX3L) complex, which results in IFN-mediated antiviral signalling and facilitating viral immune evasion [38].

Proteomic analyses revealed that the nucleocapsid (N) proteins of several coronaviruses, including SARS-CoV, MERS-CoV and murine hepatitis virus, are ADP-ribosylated, and this conserved modification is likely involving cellular PARPs. It suggests that ADP-ribosylation of the N protein represents a common host response that may influence viral RNA binding, nucleocapsid function or immune recognition during coronavirus infection [39].

One study shows that PARP7 supports murine coronavirus replication, as its knockdown reduces viral titres and enhances IFN responses [38]. IFN-inducible PARPs regulate antiviral immunity through different mechanisms. PARP14 enhances IFN-I responses by acting as a transcriptional co-regulator of STAT1-dependent gene expression, thereby increasing ISG induction. In contrast, PARP12 restricts viral replication by promoting ubiquitin-proteasome-dependent degradation of viral proteins and inhibiting their cytoplasmic translation [40]. PARP12-knockout studies confirmed its requirement for suppressing replication of macrodomain-deficient coronaviruses in a tissue-specific manner; however, incomplete rescue of viral replication suggests redundancy among other PARPs [28].

Sirtuins, particularly SIRT5 and SIRT2, also modulate coronavirus infection. SIRT5 acts as a proviral host factor that supports efficient viral replication by suppressing innate antiviral responses [41]. SIRT2 directly interacts with cGAS and promotes its deacetylation, thereby reducing its activity and limiting activation of the cGAS–STING signalling pathway. In aged mice, lower SIRT2 levels are associated with increased cGAS activation and stronger inflammatory responses during SARS-CoV-2 infection [42]. Recent work has identified SIRT1 as an important player in coronavirus infections. SIRT1 activity increases in COVID-19 patients, likely reflecting a compensatory metabolic response [43]. A yeast suppressor screen revealed that mammalian SIRT1 acts as a proviral factor for MERS-CoV, suggesting that the virus can exploit SIRT1 to support its replication [44].

CD38 also plays a crucial role in modulating both innate and adaptive immune responses during SARS-CoV-2 infection. CD38 is highly upregulated on immune cells in severe COVID-19 patients, including monocytes and T cells [45]. Viral entry via ACE2 and subsequent angiotensin II signalling activates CD38, leading to Ca²^+^ release, ROS production and downstream activation of IFN-1, NF-κB and NLRP3 pathways, contributing to cytokine storm development [46]. CD38 also affects extracellular adenosine levels by hydrolysing NAD^+^ to cADPr and ADPr, which regulate Ca²^+^ signalling and adenosine production [47]. Although the immunomodulatory effects of adenosine in COVID-19 are still being defined, recent studies have shown that SARS-CoV-2 infection disrupts the CD39/CD73 adenosine axis, characterized by increased CD39^+^ T-regulatory cells, reduced CD73 expression and diminished circulating adenosine levels, a pattern associated with impaired T-cell function [48,49].

#### Targeting NAD^+^ for therapeutic intervention

Targeting NAD^+^ metabolism and NAD^+^-consuming enzymes is being explored as a therapeutic strategy for treating coronavirus infection. Supplementation with NAD^+^ precursors such as NAM or NR has been shown to restore NAD^+^ levels and improve metabolic and immune functions. For instance, NAM administration accelerates recovery in mild-to-moderate COVID-19 by enhancing gut microbial metabolic potential [50] and improves endothelial function by reducing oxidative stress and enhancing nitric oxide production [51]. NR supplementation in K18-hACE2 mice improved recovery from SARS-CoV-2 infection, likely by restoring intracellular NAD^+^ pools [27]. Another study has shown that treatment with NAD^+^ or its precursor NMN significantly ameliorates SARS-CoV-2–induced lung inflammation and tissue damage in mouse models by restoring host metabolic and mitochondrial homeostasis [52].

A pilot trial using low-dose naltrexone plus NAD^+^ supplementation reported improvements in persistent fatigue, suggesting that supporting NAD^+^ metabolism may be beneficial in post-acute COVID-19 [53]. In an observational study of 201 hospitalized COVID-19 patients with acute kidney injury, niacinamide treatment reduced the risk of renal replacement therapy or death by ~36%, suggesting a protective effect in virus-associated kidney injury [54].

Collectively, these findings emphasize the interplay between NAD^+^ metabolism, NAD-consuming enzymes and host defence in coronavirus pathogenesis.

### Influenza virus

Influenza virus is a negative-sense virus belonging to the *Orthomyxoviridae* family. IAVs contain eight single-stranded viral RNA gene segments [55]. Extensive studies indicate that NAD^+^-related enzymes and metabolic pathways play important roles during influenza virus infection (Fig. 3b).

#### Infection-induced alterations in NAD^+^ metabolism

A recent preprint using an ex vivo mouse lung infection model showed that IAV infection rapidly remodels NAD^+^ metabolism, altering NAD^+^ consumption, modulating salvage pathway enzymes and disrupting mitochondrial NAD^+^ pools [56]. Pharmacological manipulation of NAD^+^ levels affected viral replication. Restoring NAD^+^ reduced viral titres, while NAD^+^ depletion enhanced them, suggesting NAD^+^ as a rate-limiting factor in influenza pathogenesis rather than a passive metabolic cofactor. Human population studies support this notion: reduced circulating concentrations of NAMPT and NMNAT1, key enzymes in NAD^+^ salvage and synthesis pathways, correlate with a higher risk of recurrent upper respiratory infections like IAV and IBV, indicating that compromised NAD^+^ biosynthetic capacity predisposes individuals to viral susceptibility [57].

IAV infection downregulates G6PD, leading to decreased NADPH production, impaired antioxidant defences, increased ROS accumulation and enhanced viral replication [58]. In aged lungs, influenza infection suppresses IDO1, the rate-limiting enzyme of tryptophan catabolism, resulting in defective KYNU pathway flux and diminished generation of NAD^+^ precursors [59]. Complementary work shows that IAV actively rewires KYNU metabolism, reducing NAD^+^ intermediate production and altered immunometabolism signalling that governs antiviral responses [60]. Collectively, these studies demonstrate that IAV disrupts multiple nodes of NAD^+^ metabolism.

#### Impact of NAD^+^-consuming enzymes on infection

PARPs constitute a major class of NAD^+^-consuming enzymes that profoundly influence IAV replication and host antiviral responses. PARP1, the most abundant nuclear PARP, plays a multifaceted role during IAV infection. It interacts directly with the viral polymerase complex to enhance replication efficiency and genome transcription [61], a proviral function supported by the observation that PARP1 inhibition reduces viral propagation and alleviates influenza-induced pneumonia *in vivo* [62]. PARP1 promotes proteasome-mediated degradation of the IFN-1 receptor IFNAR, thereby impairing JAK–STAT signalling and reducing ISG expression during IAV infection [63]. Although PARP1 is often considered a proviral factor during IAV infection, recent work shows that PARP1 can also act as an antiviral factor. During infection, PARP1 induces a widespread shift in host protein ADP-ribosylation patterns, generating a cellular environment that reduces viral gene expression and limits replication [64].

Beyond PARP1, other PARP family members are strategically targeted during infection. PARP10 interacts with the avian H5N1 influenza NS1 protein, resulting in PARP10 relocalization and reduced antiviral activity, enabling enhanced viral replication [65]. One study has shown that during IAV infection, PARP11 functions as a proviral factor by mono-ADP-ribosylating the E3 ubiquitin ligase component *β*-TrCP, leading to ubiquitin-mediated IFNAR1 degradation and suppression of IFN-1 signalling [66]. Constitutive aryl hydrocarbon receptor (AhR) signalling suppresses IFN-1-mediated control of IAV and is accompanied by induction of the AhR target gene PARP7 (TiPARP), supporting a role for AhR–PARP7 signalling in the suppression of antiviral immunity [67].

IAV also promotes its replication by increasing the expression of the host enzymes tankyrase 1 and tankyrase 2 (TNKS1/2) (also referred to as PARP5a/b). Elevated tankyrase activity promotes ADP-ribosylation–dependent proteasomal degradation of AXIN1, a scaffold protein that supports antiviral signalling. This results in reduced activation of JNK/c-Jun and STAT-mediated pathways, leading to suppression of IFN-1 responses [68]. In parallel, the antiviral microRNAs miR-9-1 and miR-206 directly target TNKS1 and TNKS2, respectively, lowering tankyrase expression during IAV infection [69,70]. This microRNA-mediated inhibition of tankyrases restores AXIN1-dependent antiviral signalling and strengthens the host innate immune response against influenza virus [71].

The PARP9–DTX3L complex was shown to restrict IAV infection by enhancing IFN-1 signalling. It promotes ubiquitination of host histone H2BJ and viral proteins, leading to increased ISG expression and reduced viral replication [72]. PARP12 functions as a checkpoint for necroptosis and apoptosis by MARylating key host proteins, and during influenza virus infection in mice, this activity restricts viral replication while preventing premature cell death [73]. PARP13, also known as ZAP, acts as an antiviral protein against IAV through two distinct mechanisms. The long isoform, ZAPL, contains a PARP catalytic domain that allows it to bind influenza polymerase proteins PA and PB2, marking them for proteasomal degradation and thereby limiting viral polymerase activity and replication [74]. The short isoform, ZAPS, lacks the PARP catalytic domain but still inhibits influenza by lowering viral mRNA and protein production, reducing the number of viral components available for replication [75].

Sirtuins also act as important regulators of host responses to IAV. SIRT3, a mitochondrial sirtuin, protects lung epithelial cells from IAV-induced damage by limiting oxidative stress and inflammation [76]. Overexpression or activation of SIRT3 reduces mitochondrial ROS production and inflammatory cytokine release by regulating PARP1 activity, thereby preserving mitochondrial function and cellular viability during infection.

Among nuclear sirtuins, SIRT1 modulates both metabolism and innate immunity in influenza. Compounds such as emodin and its analogues inhibit IAV replication by activating the PPARα/γ–AMPK–SIRT1 axis and reshaping fatty acid metabolism, indicating that SIRT1-dependent metabolic reprogramming can create an antiviral state [77]. Consistent with this, SIRT1 modulates innate immune responses by deacetylating key transcriptional regulators of IFN-1 and NF-κB signalling pathways, thereby influencing both antiviral defence and inflammatory responses [78]. In murine models of influenza pneumonia, pharmacological SIRT1 modulators show beneficial or detrimental effects, depending heavily on neutrophil responses. SIRT1 activity influences neutrophil recruitment, activation and tissue damage, thereby shaping the overall therapeutic outcome [79].

Cytoplasmic sirtuins also participate in influenza pathogenesis. SIRT2 has been implicated as a proviral host factor. An allosteric SIRT2 inhibitor displays broad-spectrum antiviral activity, including against IAV, by dampening SIRT2-dependent processes required for efficient viral replication [80]. SIRT2 has also been shown to regulate neutrophil functions through NAD^+^ synthesis pathways during viral infection, linking NAD^+^ metabolism, innate immune effector functions and disease severity [81]. Together, these findings show that influenza reshapes NAD^+^ metabolism and hijacks NAD^+^-consuming enzymes to control viral replication and immune responses, making NAD^+^ pathways central to disease progression.

### Human immunodeficiency virus

HIV is an enveloped, single-stranded RNA virus belonging to the family *Retroviridae*, genus *Lentivirus*. Following entry into host CD4^+^ T cells, HIV reverse-transcribes its RNA genome into DNA and integrates this proviral DNA into the host chromosomal genome, enabling persistent infection and productive viral replication [82].

#### Infection-induced alterations in NAD^+^ metabolism

**Fig. 4.**
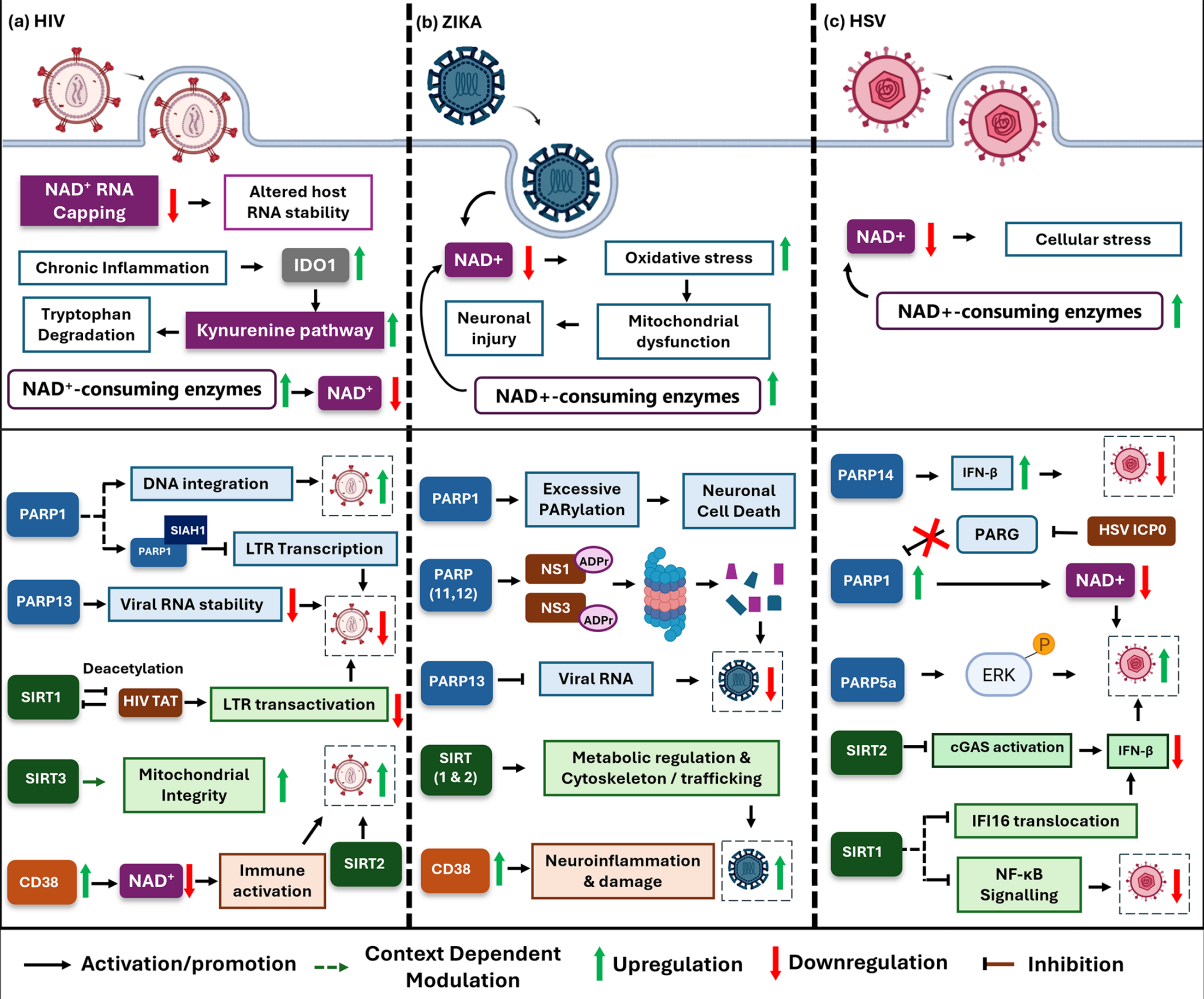
NAD^+^ metabolism and viral responses during HIV, ZIKV and HSV infection. (**a**) HIV: HIV infection enhances tryptophan degradation through activation of the KYNU pathway, contributing to immune exhaustion and a reduction in cellular NAD^+^ levels. Several PARPs regulate HIV replication: PARP1 exhibits a context-dependent role during HIV infection, inhibiting LTR-driven transcription and thereby exerting a proviral effect while also promoting viral DNA integration, which contributes to an antiviral outcome. In contrast, PARP13 restricts HIV replication by destabilizing viral RNA. SIRT1 suppresses Tat-induced HIV-1 LTR transactivation, thereby exerting an antiviral effect, but HIV Tat antagonizes SIRT1 activity to relieve this repression and promote viral transcription, whereas SIRT3 supports mitochondrial integrity. Increased CD38 expression further depletes NAD^+^ and promotes immune activation. (**b**) ZIKV: ZIKV infection leads to marked NAD^+^ depletion and oxidative stress, resulting in mitochondrial dysfunction and neuronal injury. Excessive PARP1 activation causes hyper-PARylation, contributing to neuronal cell death. PARP11 and PARP12 ADP-ribosylate ZIKV nonstructural proteins NS1 and NS3, promoting their proteasomal degradation, while PARP13 targets viral RNA; together, these mechanisms restrict viral replication. SIRT1 and SIRT2 regulate metabolic homeostasis, cytoskeletal organization and intracellular trafficking. Elevated CD38 activity exacerbates NAD^+^ loss, driving neuroinflammation and tissue damage. (**c**) HSV: HSV induces cellular stress responses that reduce NAD^+^ levels. PARP14 promotes antiviral IFN-β signalling. HSV proteins, including ICP0, inhibit poly (ADP-ribose) glycohydrolases (PARGs), leading to sustained PARP1 activity, increased NAD^+^ consumption and enhanced viral replication. Additionally, PARP5 (tankyrases) modulate ERK signalling through phosphorylation, further promoting HSV replication. SIRT2 negatively regulates the cGAS–STING DNA sensing pathway, reducing IFN-1 production and thereby promoting HSV replication. In contrast, SIRT1 acts in a context-dependent manner: it inhibits the cytoplasmic translocation of the DNA sensor IFI16, reducing IFN responses to HSV infection, and can also modulate NF-κB signalling, which may influence antiviral responses.

HIV infection profoundly disrupts host NAD^+^ metabolism, influencing cellular energy balance, immune activation and disease pathogenesis (Fig. 4a). Early work demonstrated that HIV-infected cells show a marked decrease in intracellular NAD^+^ levels, revealing a direct metabolic burden imposed by viral replication and suggesting that NAD^+^ depletion may be an early cellular stress signal in infected lymphocytes [83]. This metabolic disruption extends systemically, as NAD^+^ deficiency has been linked to tissue dysfunction, including kidney pathology in HIV-nephropathy mice where reduced NAD^+^ correlated with mitochondrial impairment and tubular injury [84], as well as reduced NAD^+^ levels in skeletal muscle, which have been linked to weakness and decreased physical strength even in asymptomatic individuals [85].

Beyond depletion, HIV also alters the biochemical fate of NAD^+^ at the RNA level. NAD^+^ capping is a recently discovered RNA modification in which the 5′ end of RNA carries an NAD^+^ instead of a canonical methylguanosine m⁷G cap, influencing RNA stability, processing and translation. HIV-1 infection significantly reduces NAD^+^ caps on host snRNAs and snoRNAs, selectively destabilizing these transcripts and potentially rewiring the nuclear RNA processing environment in favour of viral replication. By removing NAD^+^ caps, HIV-1 may selectively destabilize key host RNAs to promote a pro-viral nuclear environment [86,87].

Direct visualization using two-photon fluorescence lifetime imaging further revealed that HIV infection shortens NADH fluorescence lifetimes, indicative of a shift towards free NADH and altered mitochondrial engagement [88].

HIV also remodels core metabolic pathways such as glycolysis; latent reservoirs exhibit glycolytic downregulation, increasing oxidative stress and leaving infected cells metabolically fragile and more susceptible to death under redox pressure [89]. A major source of perturbation arises from the KYNU pathway, which is chronically activated in people with HIV. It drives tryptophan degradation towards KYNU and its metabolites rather than NAD^+^ synthesis, contributing to immune exhaustion, cognitive decline and systemic inflammation [90,92]. These alterations, combined with increased NAD^+^ consumption by PARPs and other enzymes, limit the availability of NAD^+^ precursors and result in overall NAD^+^ depletion.

#### Impact of NAD^+^-consuming enzymes on infection

NAM suppresses HIV replication in both acute and chronic models by restoring NAD^+^ pools [93], while NMN modulates T-cell activation and inflammatory signatures, ultimately influencing viral infection dynamics [94].

PARP1 plays multiple, context-dependent roles in HIV-1 infection, influencing integration, transcriptional regulation and viral latency. PARP1 is required for efficient HIV-1 integration in human CD4^+^ T cells [95], helping position the provirus near preferred genomic regions such as centromeric alphoid DNA [96], although this requirement is not universal across species, as murine retroviral infection can proceed without PARP1 [97]. Beyond integration, PARP1 also shapes transcriptional outcomes: it can function as a transcriptional repressor of integrated retroviruses [98], inhibit HIV-1 transcription by competitively binding to TAR RNA and limiting Tat–p-TEFb–mediated elongation [99] and suppress LTR-driven gene expression via the PARP1–Siah1 regulatory axis [100]. Conversely, PARP1 can also act in the opposite direction by supporting certain aspects of HIV-1 replication, as demonstrated by RNAi-mediated PARP1 knockdown, which markedly reduces viral replication in human cells [101]. Pharmacological targeting of the enzyme has revealed dual functional outcomes: PARP1 inhibition not only suppresses HIV-1 LTR activity but also decreases Rho GTPase signalling, thereby impairing viral replication [102]; moreover, combining PARP inhibitors with HDAC inhibitors enhances reactivation and targeting of latent HIV reservoirs [103].

Another member of the PARP family, PARP13 (ZAP), is a catalytically inactive but potent antiviral RNA-binding protein that restricts HIV-1 by selectively degrading multiply spliced viral mRNAs [104]. This restriction is strengthened when the viral genome contains elevated CpG dinucleotide content, enabling host recognition of non-self RNA [105], and requires the cofactor KHNYN for full antiviral function [106].

Sirtuins play diverse and context-dependent roles in HIV-1 infection, with SIRT1 being the most extensively characterized. SIRT1 regulates HIV transcription by deacetylating Tat and components of the NF-κB pathway, thereby suppressing Tat-induced LTR transactivation [107], while HIV-1 Tat directly inhibits SIRT1 catalytic activity through physical interaction [108]. Pharmacologic activation of SIRT1 with resveratrol reverses this inhibition and reduces Tat-driven LTR activity in a NAD^+^-dependent manner [109]. Tat’s effects are further linked to the NAMPT–SIRT1 axis, which modulates cellular NAD^+^ availability and LTR transactivation [110]. Inhibiting SIRT1 leads to activation of the p53 pathway and increased cellular stress [111], and broader reviews highlight SIRT1’s roles in HIV latency, immune activation, metabolic control and T-cell homeostasis [112,113].

Beyond SIRT1, other sirtuins also contribute to HIV pathogenesis. SIRT3 protects mitochondrial integrity by limiting HIV-Tat–induced oxidative stress and microglial senescence [114]. SIRT2 enhances HIV replication and reactivation potential and is elevated in individuals with higher viral load and neurological dysfunction [115]. SIRT6 expression is altered during HIV infection and ART, together with SIRT1 and SIRT3, reflecting dynamic regulation of nuclear and mitochondrial sirtuins during disease progression [116]. Nuclear sirtuins such as SIRT6 and SIRT7 also modulate chromatin architecture and influence HIV LTR transcription [113].

The ectoenzyme CD38 influences both HIV-1 replication and disease progression by coupling immune activation to NAD^+^ metabolism. Early mechanistic work showed that engagement or overexpression of human CD38 can enhance the early steps of the HIV-1 replication cycle, including viral entry and reverse transcription, suggesting a direct proviral role in infected or bystander lymphocytes [117,118]. CD38 is more than a mere activation marker, proposing that it acts as an active player in virus–host interactions, potentially facilitating infection and propagation in CD4^+^ T cells [119]. Beyond this, CD38 regulates immune responses through its NAD^+^ hydrolase activity, leading to depletion of intracellular NAD^+^ and modulation of metabolic and signalling pathways that influence T cell activation and cytokine production. Persistent CD38 overexpression has been proposed to contribute to HIV disease progression by sustaining chronic immune activation and perturbing the CD38–NAD^+^ axis [120]. In addition, CD38 can exert immunosuppressive effects during chronic viral infection by promoting CD8^+^ T cell exhaustion, likely through NAD^+^ depletion and associated metabolic dysfunction, thereby impairing antiviral effector function and facilitating viral persistence [121].

Overall, HIV profoundly disrupts NAD metabolism, linking viral replication to altered immunity and cellular stress, and revealing NAD^+^-dependent pathways as potential therapeutic targets.

### Zika virus

ZIKV is a mosquito-borne flavivirus belonging to the family *Flaviviridae. Aedes* mosquitoes, primarily *Aedes aegypti* and *Aedes albopictus*, are the main vectors responsible for transmission to humans [122].

#### Infection-induced alterations in NAD^+^ metabolism

ZIKV profoundly disrupts host NAD^+^ metabolism, particularly in neural tissues, where metabolic stress contributes directly to viral replication and disease pathology (Fig. 4b). A study demonstrated that ZIKV infection in the developing brain causes widespread alterations in metabolic pathways, including oxidative phosphorylation, glycolysis and notably NAD^+^ biosynthesis and consumption, leading to substantial reductions in NAD^+^ levels that correlate with microcephaly phenotypes [123]. Complementary work in human foetal neuronal progenitors shows that ZIKV induces marked shifts in cellular metabolism affecting mitochondrial function, redox homeostasis and energy production in ways that can impair early neurodevelopment and reinforce the vulnerability of NAD^+^-dependent processes during infection [124]. ZIKV replication exhibits a strong dependence on redox balance and NAD(H)-linked reactions as ZIKV elevates oxidative stress and perturbs intracellular NAD(H) pools [125].

#### Impact of NAD^+^-consuming enzymes on infection

Activation of the Nrf2 antioxidant pathway or treatment with NAD(H) antimetabolites can significantly suppress ZIKV viral replication by restoring redox equilibrium or disrupting NAD^+^-dependent metabolic steps essential for the viral life cycle [125].

Several members of the PARP family play critical but distinct roles during ZIKV infection. ZIKV infection directly activates PARP1, leading to excessive poly-ADP-ribosylation, rapid depletion of cellular NAD^+^ pools and PARP1-mediated cell death, a mechanism that contributes to neuronal loss and may be responsible for ZIKV-associated neurodevelopmental defects [126]. In contrast to this pathogenic role, IFN-stimulated mono-ADP-ribosyltransferases PARP12 and PARP11 function as potent antiviral restriction factors against ZIKV. PARP12 suppresses viral replication by promoting PARP-dependent degradation of the viral nonstructural proteins NS1 and NS3, thereby directly limiting viral RNA replication and protein stability [127]. PARP11 acts synergistically with PARP12 to enhance this antiviral response, further restricting ZIKV replication through coordinated ADP-ribosylation–dependent mechanisms [128]. In addition to enzymatically active PARPs, PARP13 contributes to host defence by binding viral RNAs and promoting their degradation. Characterization of ZAP splice variants suggests functional diversity that may influence antiviral potency against flaviviruses, including ZIKV [129].

ZIKV replication also depends on host sirtuin activity. Chemical screening studies identified Tenovin-1, an inhibitor of SIRT1 and SIRT2, as a potent antiviral against ZIKV, demonstrating that pharmacological inhibition of sirtuins significantly reduces viral replication. Mechanistic analyses suggested that Tenovin-1 disrupts sirtuin-regulated metabolic and cytoskeletal processes required for efficient viral replication rather than directly targeting viral enzymes [130,131]. Consistently, allosteric inhibition of SIRT2 using FLS-359 also exhibited broad-spectrum antiviral activity and impaired ZIKV replication, implicating SIRT2-dependent regulation of intracellular trafficking and microtubule dynamics in the ZIKV life cycle [80].

Recent evidence identifies the ectoenzyme CD38 as a key mediator of NAD^+^ depletion during ZIKV infection, particularly in the brain. In ZIKV-infected mice, CD38 expression is markedly upregulated in neural tissues, coinciding with significant reductions in NAD^+^ levels. Genetic deletion or pharmacological inhibition of CD38 preserves brain NAD^+^ pools, reduces inflammatory responses and mitigates neuronal damage, indicating that CD38-driven NAD^+^ consumption contributes directly to ZIKV-associated neuropathology [132]. Overall, ZIKV disrupts host NAD^+^ metabolism to support replication and drive neurodevelopmental injury.

### Herpes simplex viruses

HSV-1 and HSV-2 are enveloped double-stranded DNA viruses of the family *Herpesviridae* (subfamily *Alphaherpesvirinae*) that establish lifelong latency in sensory neurons [133].

#### Infection-induced alterations in NAD^+^ metabolism

HSV-1 infection induces marked alterations in host NAD^+^ metabolism that influence viral replication and neuropathology (Fig. 4c). HSV-1 activates cellular stress responses that drive rapid NAD^+^ consumption and metabolic imbalance during infection [134,135]. Recent studies have identified NAMPT as an IFN-inducible restriction factor against HSV-1, acting through a non-canonical protein phosphoribosylase activity that limits virion protein incorporation rather than classical NAD^+^ biosynthesis [136]. Another study shows that the microbiota-derived NAD^+^-related metabolite nicotinamide N-oxide (NAMO) protects against herpes simplex encephalitis by activating mitophagy in microglia and suppressing neuroinflammation [137]. HSV-1 replication is also sensitive to redox regulation, as upregulation of Nrf2-dependent antioxidant responses significantly represses viral replication, linking NAD^+^-associated redox balance to HSV-1 pathogenesis [138].

#### Impact of NAD^+^-consuming enzymes on infection

PARP family enzymes are central regulators of HSV-1 infection, functioning at the intersection of viral replication, innate immunity and NAD^+^ consumption. IFN-induced PARPs such as PARP14 enhance antiviral immunity by promoting IFN production and modulating replication of multiple viruses, including HSV-1 [139]. Early work demonstrated that HSV immediate-early proteins undergo poly ADP-ribosylation, implicating PARPs directly in viral gene regulation [134]. HSV-1 infection robustly activates PARP-1, triggering extensive PAR synthesis and ICP0-dependent degradation of PARG, thereby sustaining PARP activity and exacerbating NAD^+^ depletion [135]. Functional studies further showed that HSV-1 requires PARP activity for efficient replication, in part through ERK-dependent phosphorylation and ICP0-mediated nuclear localization of tankyrase-1 (PARP5a) [140].

Sirtuins, as NAD^+^-dependent deacetylases, play multifaceted roles in HSV-1 infection by regulating innate immune sensing, inflammation and neuronal survival. SIRT2 negatively regulates the cGAS–STING signalling pathway by deacetylating G3BP1, a scaffold protein required for efficient cGAS activation. This disrupts cGAS phase separation and attenuates downstream STING–TBK1–IRF3 signalling, leading to reduced IFN-1 responses during DNA virus infection such as HSV 1 [141]. SIRT1 negatively regulates antiviral responses by preventing cytoplasmic translocation of IFI16, thereby dampening DNA sensing during HSV-1 infection [142]. HSV-1 replication and neuronal injury are closely linked to host energy-sensing pathways, as modulation of the AMPK–SIRT1 axis suppresses viral replication and protects neurons from HSV-induced neurodegeneration [143,144]. In microglia, nicotinamide N-oxide attenuates HSV-1–induced inflammation through SIRT1-dependent inhibition of NF-κB signalling, reinforcing the protective role of SIRT1 in neuroinflammatory contexts [145]. Collectively, HSV-induced modulation of NAD^+^-dependent pathways highlights an important metabolic dimension of virus–host interactions.

## Discussion/conclusion

Viruses across diverse families, including SARS-CoV-2, IAV, HSV, ZIKA and HIV, have evolved distinct strategies to perturb host NAD^+^ metabolism in ways that support replication and facilitate immune evasion. These strategies largely involve altering the balance between NAD^+^ biosynthesis and consumption, as well as modulating the activity of key NAD^+^-dependent enzymes such as PARPs, CD38 and sirtuins. While NAD^+^-consuming enzymes, particularly PARPs, can exert antiviral functions, their sustained or excessive activation may deplete intracellular NAD^+^ pools and contribute to inflammation and cell death. HIV and ZIKA, for example, engage NAD^+^-regulated pathways in virus-specific contexts associated with viral latency and neurotropism, respectively, underscoring the diversity of NAD^+^ modulation across infections. Interventions aimed at restoring or stabilizing NAD^+^ levels through precursor supplementation or targeted enzyme inhibition have shown promise in counteracting virus-induced metabolic dysregulation. However, the dual roles of NAD^+^-regulating enzymes in both host defence and viral facilitation highlight the need for context- and virus-specific investigation. Notably, it remains unclear in several contexts whether these antiviral or proviral effects are strictly dependent on enzymatic activity or also involve non-catalytic functions, such as transcriptional co-regulation (e.g. PARP14) or scaffolding roles (e.g. PARP9–DTX3L complex).

Recent studies further indicate that the regulatory landscape of NAD^+^ metabolism in immunity extends beyond these canonical pathways. Non-canonical SIR domain–containing proteins, including SIRal (FAM118B) and SIRal2, have recently been implicated in innate immune signalling, particularly in association with Toll-like receptor pathways, and may function as previously unrecognized modulators of NAD^+^ homeostasis during infection. Although their enzymatic properties and antiviral roles are still being defined, their structural similarity to sirtuins combined with emerging functional data suggests that NAD^+^ consumption may be more broadly embedded within immune signalling networks than previously appreciated [146,147]. In parallel, SARM1 represents a mechanistically distinct NAD^+^-consuming enzyme whose intrinsic TIR-domain NADase activity enables rapid NAD^+^ cleavage. Although best characterized in the context of axonal degeneration, SARM1 is evolutionarily linked to innate immune signalling pathways, and TIR-domain–mediated NAD^+^ depletion has emerged as a conserved mechanism of host defence. In this context, SARM1 may function as a metabolic checkpoint that couples immune activation to NAD^+^ consumption, although its direct antiviral roles in mammalian systems remain to be fully defined [148,149].

This broader perspective is supported by work in bacterial and plant systems, where NAD^+^ depletion has been identified as a key antiviral defence mechanism. In these contexts, host pathways actively reduce NAD^+^ levels to restrict pathogen replication, while bacteriophages have evolved countermeasures to restore or maintain NAD^+^ pools. This interplay reflects an ongoing host–pathogen arms race centred on NAD^+^ homeostasis. Although similar mechanisms have not yet been clearly established in mammalian viral infections, these findings raise the possibility that viruses may also engage strategies to modulate NAD^+^ availability at a systems level [150]. Ultimately, a deeper understanding of NAD^+^ biology across viral infections will be critical for the rational development of effective host-directed antiviral therapies.

## Abbreviations

ACMS: *α*-amino-*β*-carboxymuconate *ε*-semialdehyde
ADPr: ADP-ribose
AhR: aryl hydrocarbon receptor
cADPr: cyclic ADP-ribose
DTX3L: deltex E3 ubiquitin ligase 3-like protein
G6PD: glucose-6-phosphate dehydrogenase
3-HAA: 3-hydroxyanthranilic acid
HIV: human immunodeficiency virus
HSV: herpes simplex virus
IAV: influenza A virus
*IDO1*: indoleamine 2,3-dioxygenase 1
IFN-I: Type I interferon
ISG: interferon-stimulated gene
KYNU: kynurenine
LDHB: lactate dehydrogenase B
N: nucleocapsid
NA: nicotinic acid
NAAD: nicotinic acid adenine dinucleotide
NAD^+^: nicotinamide adenine dinucleotide
NADP: nicotinamide adenine dinucleotide phosphate
NAM: nicotinamide
NAMN: nicotinic acid mononucleotide
NAMPT: nicotinamide phosphoribosyl transferase
NAPRT: nicotinic acid phosphoribosyl transferase
NMN: nicotinamide mononucleotide
NMNAT1-3: nicotinamide mononucleotide adenylyl transferases 1–3
NR: nicotinamide riboside
Nsp3: nonstructural protein 3
PARGs: poly (ADP-ribose) glycohydrolases
PARPs: poly (ADP-ribose) polymerases
ROS: reactive oxygen species
TNKS1: tankyrase 1
TNKS2: tankyrase 2
ZIKV: Zika virus
